# Assessing cumulative dose distributions in combined radiotherapy for cervical cancer using deformable image registration with pre-imaging preparations

**DOI:** 10.1186/s13014-014-0293-4

**Published:** 2014-12-20

**Authors:** Takanori Abe, Tomoaki Tamaki, Souichi Makino, Takeshi Ebara, Ryuuta Hirai, Kazunori Miyaura, Yu Kumazaki, Tatsuya Ohno, Naoto Shikama, Takashi Nakano, Shingo Kato

**Affiliations:** Department of Radiation Oncology, Saitama Medical University, International Medical Center, 1397-1 Yamane, Hidaka-shi, 350-1298 Japan; Gunma Prefectural Cancer Center, 617-1 Takabayashinishi-machi, Ohta-shi, Gunma 373-8550 Japan; Gunma University Heavy Ion Medical Center, 3-39-22 Showa-machi, Maebashi-shi, Gunma 371-8511 Japan; Department of Radiation Oncology, Gunma University, 3-39-22 Showa-machi, Maebashi-shi, Gunma 371-8511 Japan

**Keywords:** Radiotherapy, Cervical cancer, Deformable image registration

## Abstract

**Background:**

The purpose of the study was to evaluate the feasibility of deformable image registration (DIR) in assessing cumulative dose distributions of the combination of external beam radiotherapy (EBRT) and fractionated intracavitary brachytherapy (ICBT) for cervical cancer.

**Materials and methods:**

Three-dimensional image data sets of five consecutive patients were used. The treatment plan consisted of whole pelvic EBRT (total dose: 45 Gy in 25 fractions) combined with computed tomography (CT)-based high-dose rate ICBT (≥24 Gy in 4 fractions to the high risk clinical target volume (HR-CTV)). Organs at risk and HR-CTV were contoured on each CT images and dose-volume parameters were acquired. Pre-imaging preparations were performed prior to each ICBT to minimize the uncertainty of the organ position. Physical doses of each treatment were converted to biologically equivalent doses in 2 Gy daily fractions by the linear quadratic model. Three-dimensional dose distributions of each treatment were accumulated on CT images of the first ICBT using DIR with commercially available image registration software (MIM Maestro®). To compare with DIR, 3D dose distributions were fused by rigid registration based on bony structure matching. To evaluate the accuracy of DIR, the Dice similarity coefficient (DSC) was measured between deformed contours and initial contours.

**Results:**

The cumulative dose distributions were successfully illustrated on the CT images using DIR. Mean DSCs of the HR-CTV, rectum, and bladder were 0.46, 0.62 and 0.69, respectively, with rigid registration; and 0.78, 0.76, and 0.87, respectively, with DIR (p <0.05). The mean DSCs derived from our DIR procedure were comparable to those of previous reports describing the quality of DIR algorithms in the pelvic region. DVH parameters derived from the 2 methods showed no significant difference.

**Conclusions:**

Our results suggest that DIR-based dose accumulation may be acceptable for assessing cumulative dose distributions to assess doses to the tumor and organs at risk in combined radiotherapy for cervical cancer under pre-imaging preparations.

## Background

The combination of external beam radiotherapy (EBRT) and intracavitary brachytherapy (ICBT) is the standard treatment for cervical cancer [1]. In conventional ICBT, two orthogonal X-rays are taken and “point A” is used as the reference point for dose prescription. “Rectal and bladder points” have also been used as reference points for the assessment of rectal and bladder doses, according to Report 38 of the International Commission on Radiation Units and Measurements [2]. However, these points are hypothetical, and do not always represent the actual tumor volume or the exact locations of the highest doses in the rectum and bladder [3,4]. Recently, three-dimensional (3D) image modalities, such as computed tomography (CT) and magnetic resonance imaging (MRI), have become available for use in planning ICBT treatment. Treatment planning based on 3D images allows for assessment of 3D dose distributions and dose-volume evaluation. The Group Européen de Curiethérapie-European Society for Radiotherapy and Oncology (GEC-ESTRO) working group for gynecologic brachytherapy has provided recommendations for 3D image-based treatment planning in cervical cancer brachytherapy [5-7]. Several studies have demonstrated that 3D dose-volume parameters of the target volume and organs at risk (OARs) are useful for predicting treatment response and toxicity development [8-12].

For assessment of cumulative dose-volume relationships for the target volumes and OARs in the combined radiotherapy, the GYN GEC-ESTRO working group suggests several parameters, including D90 of HR-CTV, and D2cc of the rectum and bladder. These 3D dose-volume histogram (DVH) parameters are calculated by simply adding DVH parameters for the target volume and OARs in EBRT for each ICBT session. However, simple addition of DVH parameters is based on the assumption that the location of the region of interest is identical in each therapy. If the high dose area is not consistent in each therapy, simple addition of DVH parameters does not reflect the absolute dose-volume relationship. Under such circumstances, it is necessary to illustrate cumulative dose distributions and calculate DVH in order to estimate the dose-volume relationship. However, assessing cumulative dose distributions with a conventional treatment planning system is difficult due to the following reasons. First, as EBRT and ICBT have different dose profiles and fractionations, it is generally difficult to calculate DVH [13]. Second, organ motions of the uterus, bladder, and rectum, and changes in target volume during treatment, are significant during cervical cancer treatment [14]; therefore dose accumulation with conventional rigid registration is inaccurate and unreliable.

Recently, a few studies have demonstrated the efficacy of DIR for evaluating cumulative dose distributions and DVHs in combined radiotherapy for various tumors [15-17]. Therefore, we aimed to conduct a pilot study to evaluate the feasibility of DIR for assessment of cumulative dose distributions and DVHs of combined radiotherapy for cervical cancer using a commercially available DIR algorithm (MIM Maestro®).

## Methods

### Patients

Data were collected from five consecutive patients with locally advanced cervical cancer, who were treated with EBRT and high-dose rate (HDR)-ICBT in our institution between August and October 2013. All the data collections were performed after the approval of institutional review board and acquiring written informed consent from patients.

### Treatment

EBRT was delivered to the whole pelvis with a total dose of 45 Gy in 25 fractions using the four-field box technique with 10-MV X-ray. The clinical target volume (CTV) of EBRT consisted of the cervical tumor, whole uterus, bilateral parametria, at least the upper half of the vagina, and pelvic lymph nodes (including the common, external, and internal iliac, and presacral lymph nodes). The planned target volume (PTV) of EBRT included the CTV plus a ≥10-mm safety margin.

High-dose-rate (HDR) ICBT was performed weekly for four consecutive weeks using ^192^Ir sources. A combination of tandem and ovoid applicators was used. CT-based 3D image guided brachytherapy was performed for every session of ICBT. A series of transverse CT images with 1 mm slice thickness were obtained with the applicators in place. The high risk CTV (HR-CTV), rectum, and bladder were delineated according to GEC-ESTRO recommendations [6]. For precise delineation, references were always made to the MRI at diagnosis and those obtained within a week prior to the first brachytherapy session. The minimum dose delivered to 90% of the most irradiated volume of HR-CTV (HR-CTV D90) and the minimum doses delivered to 2 cm^3^ (D2cc) of the most irradiated volumes of the rectum and the bladder were calculated and recorded. At least 6 Gy was prescribed to HR-CTV D90 in each ICBT session. Dose constraint was 75 Gy_EQD2_ in D2cc of the rectum. Dose prescription and target coverage were modified based on dose constraints for OARs.

### Pre-imaging preparations for deformable image registration

Pre-imaging preparations were carefully performed before every ICBT session, as large variation in organ position and volume may produce fusion uncertainties and affect the quality of DIR [18,19]. Patients were required to collect urine one hour before undergoing CT imaging for EBRT treatment planning. Bladder volume was confirmed by this CT image. In every ICBT session, the bladder was filled with this volume of normal saline to stabilize its size and position. The original angle and length of the longitudinal axis of the uterus were also confirmed by treatment planning CT for EBRT. In all ICBT sessions, the angle of uterus was kept constant by adjusting the inserting angle of tandem applicator. Patients were required to defecate before any treatment. Distension of the rectum was confirmed by treatment planning CT for EBRT and brachytherapy. If gas was observed in the rectum at ICBT, gas drainage was performed to reduce rectal distention. CT was retaken if gas drainage was performed.

### Dose accumulation

**Figure 1:**
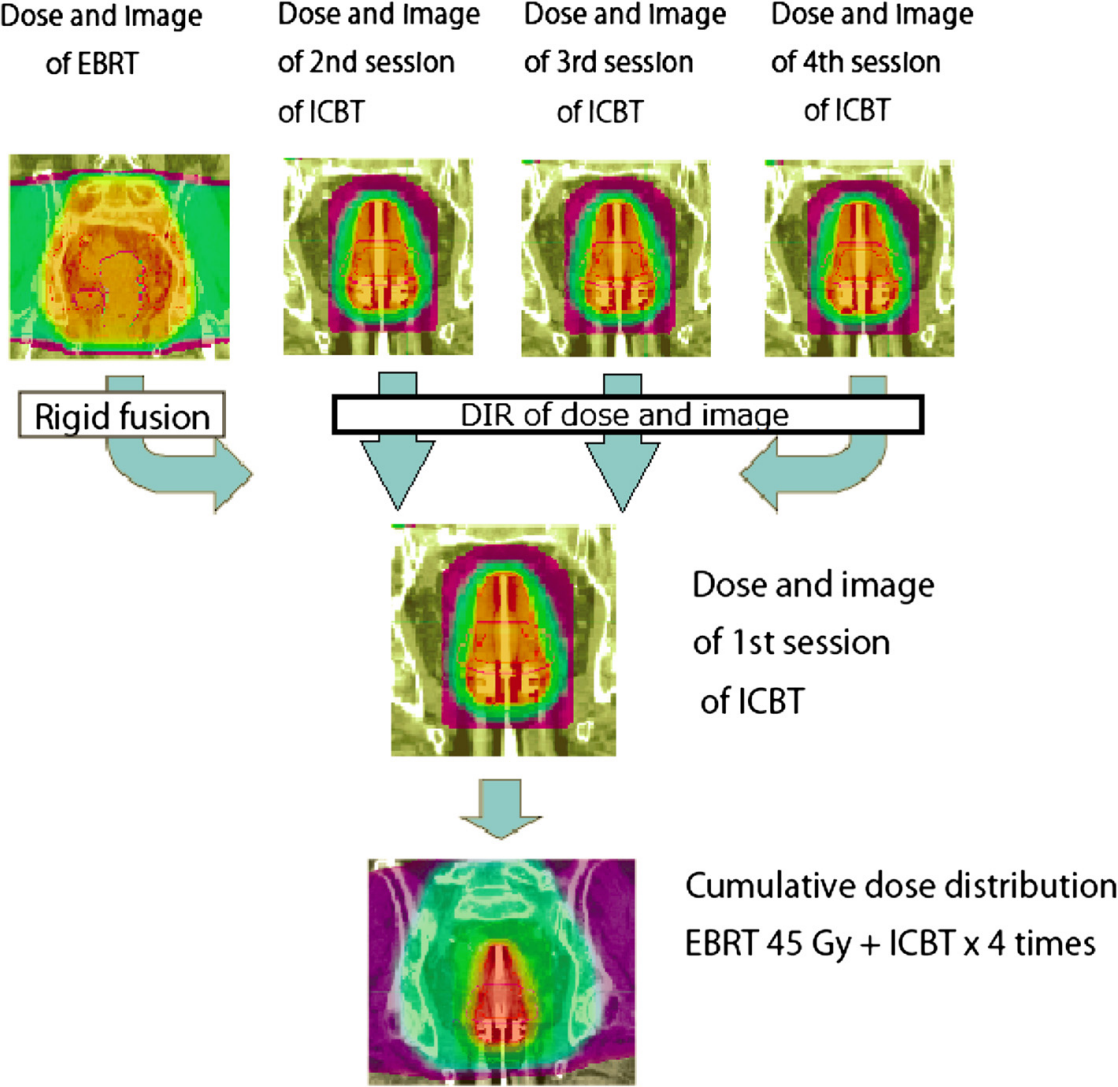
**The flow chart of dose accumulation.** Doses of radiotherapy were converted to biologically equivalent doses in 2 Gy per fraction using the linear quadratic model with α/β = 10 Gy for tumor tissues and α/β = 3 Gy for normal tissues. First, dose and image of EBRT were rigidly fused on the CT image of the first ICBT. Then, using DIR, doses and images of the second, third, and fourth ICBT were accumulated on those of the first ICBT.

In all cases, image registration was performed using MIM maestro ver.6.2 (MIM Software Inc., Cleveland, OH, USA). Doses of radiotherapy were converted into biologically equivalent doses in 2 Gy daily fractions (Gy_EQD2_) using the linear quadratic model with α/β = 10 Gy for tumor tissues, and α/β = 3 Gy for normal tissues [20]. At first, CT image data sets of EBRT were rigidly fused by matching bony structures on CT images of the first ICBT. Next, CT image data sets of the second, third, and fourth ICBT sessions were rigidly fused, referring to the applicator position in the CT images of the first ICBT. Finally, DIR was performed to accumulate doses of the second, third, and fourth ICBT on the first ICBT dose (Figure 1). Results of the DIR were carefully reviewed using the function of MIM maestro (Reg Review) and modified by another function (Reg Refine).

### Cumulative dose-volume evaluations

Cumulative DVHs of the HR-CTV, rectum, and bladder were calculated based on accumulated dose distributions. DIR-based DVH parameters such as HR-CTV D90, D2cc of the rectum and bladder were derived from cumulative DVHs.

HR-CTV D90 and D2cc of the rectum and bladder were also calculated using conventional simple addition of DVH parameters. In this method DVH parameters were calculated by adding the components of EBRT and ICBT sessions.

### Quantitative evaluation of DIR performance

Although no established method exists for estimating the quality of DIR, the Dice similarity coefficient (DSC) is commonly used to evaluate the accuracy of DIR [18,21,22], DSC was calculated by the following formula [23]:

$$ DSC=\frac{2\left({V}_{\mathrm{DIR}}\cap {V}_{\mathrm{initial}}\right)}{V_{\mathrm{DIR}}+{V}_{\mathrm{initial}}} $$

In this formula, V_DIR_ represents the volume of deformed contour after DIR, and V_initial_ represents the volume of contours manually delineated on the CT image of the first ICBT session. The DSC is used to evaluate the spatial overlap accuracy of automated segmentation of images. Values for DSC range from 0 to 1, with higher values indicating larger volumes of overlap between two images. DSC is also calculated to estimate overlap of the contours in case of rigid fusion.

### Statistical analysis

DIR-based cumulative DVH parameters and simple DVH parameter addition were compared using the paired t-test. All statistical analyses were performed using IBM SPSS Statistics for Windows, Version 21.0 (SPSS Inc., Armonk, NY, USA). p < 0.05 was considered statistically significant.

## Results

### Patient characteristics

Among the 5 patients included in the study, 3 had Stage IIB disease and 2 had Stage IIIB disease. The mean transverse tumor diameter was 5.2 cm at diagnosis and 3.8 cm at the initiation of ICBT.

### Performance of DIR

**Figure 2:**
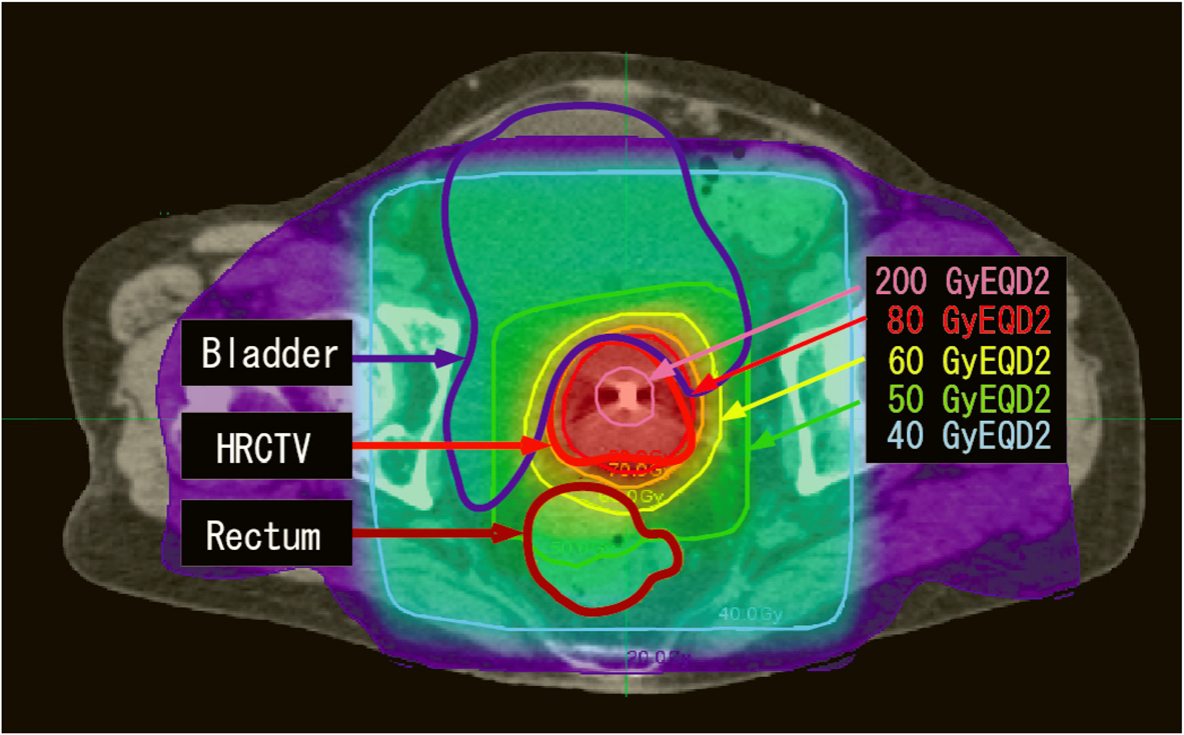
**Cumulative dose distribution.** A large dose gradient was observed in the irradiated field. There is an area with a very high dose at the center of the tumor. HR-CTV was covered by a 70–80 GyEQD2 isodose line. Pelvic lymph node areas were covered by 40–50 GyEQD2 isodose lines, depending on their distances from the central axis of the uterus.

Cumulative dose distributions, consisting of EBRT and four sessions of ICBT, were successfully illustrated using DIR (Figure 2). Inspection by 3 radiation oncologists (TA, TT, SK) revealed that dose distributions were illustrated without any irregular dose warp or irresponsible isodose lines. A high dose area (>200 Gy_EQD2_) was observed around the center of the tumor and steep dose decline was observed toward the peripheral area of the tumor. HR-CTV was covered with 70–80 Gy_EQD2_. The parametria were covered with 50–60 Gy_EQD2_. External iliac, internal iliac, and obturator lymph nodes were covered with 40–50 Gy_EQD2_ depending on the distance from the uterus. The doses to the rectum were approximately 60–70 GyEQD2.

The mean DSCs for the HR-CTV, rectum, and bladder were 0.46, 0.62, and 0.69, respectively, with rigid fusion, and 0.78, 0.76, and 0.87, respectively, with DIR. Volumetric parameters related to our DIR procedure and DSCs are shown in Tables 1, 2 and 3. The difference in DSCs between the two methods was statistically significant, suggesting the improved spatial overlap accuracy of automated image segmentation with DIR.

**Table 1:** **Volume of the original contour and deformed contour and the results of dice similarity coefficient (HR-CTV)**

		**Original contour (cm** ^**3**^ **)**	**Deformed contour (cm** ^**3**^ **)**	**Original contour ⋂ deformed contour (cm** ^**3**^ **)**	**DSC by DIR**	**DSC by rigid fusion**
Case1	DIR1	25.4	29.9	25.4	0.79	0.70
	DIR2	-	20.5	16.4	0.71	0.49
	DIR3	-	20.0	16.6	0.73	0.57
	Mean				0.75	0.59
Case2	DIR1	17.8	20.6	15.3	0.80	0.35
	DIR2		18.0	13.4	0.75	0.69
	DIR3		18.1	13.4	0.75	0.14
	Mean				0.76	0.39
Case3	DIR1	40.0	48.7	36.5	0.82	0.25
	DIR2		32.8	26.8	0.73	0.46
	DIR3		33.7	27.7	0.75	0.30
	Mean				0.77	0.34
Case4	DIR1	18.1	21.4	16.5	0.83	0.66
	DIR2	-	13.1	10.9	0.70	0.48
	DIR3	-	17.6	12.5	0.70	0.42
	Mean				0.74	0.52
Case5	DIR1	27.5	24.0	20.8	0.85	0.47
	DIR2	-	27.5	21.8	0.83	0.53
	DIR3	-	24.8	21.7	0.88	0.43
	Mean				0.86	0.48
Total	Mean ± SD				0.78 ± 0.06	0.46 ± 0.16 p <0.05

**Table 2:** **Volumes of the original and deformed contours and dice similarity coefficients (rectum)**

		**Original contour (cm** ^**3**^ **)**	**Deformed contour (cm** ^**3**^ **)**	**Original contour ⋂ deformed contour (cm** ^**3**^ **)**	**DSC by DIR**	**DSC by rigid fusion**
Case1	DIR1	47.8	40.9	32.4	0.73	0.72
	DIR2	-	26.9	24.6	0.74	0.66
	DIR3	-	50.2	33.3	0.68	0.62
	Mean				0.72	0.66
Case2	DIR1	46.6	54.1	42.5	0.85	0.74
	DIR2	-	55.3	42.4	0.80	0.73
	DIR3	-	48.1	40.7	0.75	0.67
	Mean					0.71
Case3	DIR1	46.7	53.7	32.5	0.72	0.60
	DIR2	-	47.0	29.6	0.71	0.70
	DIR3	-	79.3	39.5	0.62	0.57
	Mean				0.68	0.62
Case4	DIR1	95.4	59.9	56.7	0.76	0.52
	DIR2	-	77.6	68.5	0.71	0.51
	DIR3	-	75.7	69.0	0.84	0.41
	Mean				0.77	0.48
Case5	DIR1	51.0	51.7	40.1	0.76	0.73
	DIR2	-	36.9	31.4	0.74	0.50
	DIR3	-	38.4	33.4	0.80	0.62
	Mean				0.77	0.61
Total	Mean ± SD				0.76 ± 0.05	0.62 ±0.10 p <0.05

**Table 3:** **Volumes of the original and deformed contours and dice similarity coefficients (bladder)**

		**Original contour (cm** ^**3**^ **)**	**Deformed contour (cm** ^**3**^ **)**	**Original contour ⋂ deformed contour (cm** ^**3**^ **)**	**DSC by DIR**	**DSC by rigid fusion**
Case1	DIR1	191.2	237.9	178.2	0.86	0.83
	DIR2	-	138.1	132.0	0.85	0.84
	DIR3	-	276.4	177.7	0.88	0.82
	Mean				0.86	0.83
Case2	DIR1	463.7	392.4	365.2	0.89	0.53
	DIR2	-	434.6	121.0	0.92	0.54
	DIR3	-	370.3	350.6	0.90	0.50
	Mean				0.86	0.52
Case3	DIR1	231.7	411.3	223.6	0.89	0.79
	DIR2	-	540.3	386.0	0.59	0.48
	DIR3	-	213.9	229.4	0.93	0.82
	Mean				0.80	0.70
Case4	DIR1	337.7	204.5	196.0	0.90	0.57
	DIR2	-	184.7	171.5	0.91	0.60
	DIR3	-	313.5	302.1	0.93	0.72
	Mean				0.91	0.63
Case5	DIR1	155.9	143.0	134.2	0.89	0.77
	DIR2	-	156.2	141.1	0.91	0.83
	DIR3	-	207.9	148.8	0.80	0.70
	Mean					0.77
Total	Mean±SD				0.87 ± 0.09	0.69± 0.14 p <0.05

### DVH Parameters

The mean HR-CTV D90 and D2cc of the rectum and bladder were 81.4 Gy_EQD2_, 65.7 Gy_EQD2_, and 82.8 Gy_EQD2_, respectively, with DIR; and 83.1 Gy_EQD2_, 67.2 Gy_EQD2_, and 86.6 Gy_EQD2_, respectively, with conventional simple DVH parameter addition. There was no statistically significant difference in the dosimetric parameters between the two calculation methods (Table 4). DVH parameters calculated with DIR were comparable with those derived by the conventional method, when the anatomical locations of the uterus, rectum, and bladder were adequately matched.

**Table 4:** **Dose-volume histogram (DVH) parameters**

		**Cumulative DVH parameter**	**Simple DVH parameter addition**
HR-CTV D90	Case 1	76.4	79.0
	Case 2	84.3	82.9
	Case 3	79.4	82.5
	Case 4	82.1	85.6
	Case 5	84.9	85.5
	Mean ± SD	81.4 ± 3.5	83.1 ± 2.7 p = 0.424
Rectum D2cc	Case 1	67.8	72.2
	Case 2	58.0	58.2
	Case 3	57.9	62.1
	Case 4	74.9	74.1
	Case 5	69.7	69.4
	Mean ± SD	65.4± 8.3	67.2 ± 6.8 p = 0.719
Bladder D2cc	Case 1	65.9	74.2
	Case 2	85.2	91.4
	Case 3	80.2	84.7
	Case 4	104.2	102.9
	Case 5	78.4	79.7
	Mean ± SD	83.6 ± 11.7	86.6 ± 11.1 p = 0.687

## Discussion

Assessing cumulative dose distributions in combined therapy with EBRT and ICBT for cervical cancer has been challenging due to the difficulty in combining different types of fractionated radiation therapy, organ motion uncertainty, and tumor volume regression during treatment. Dose distributions provided by the DIR appeared to represent the characteristic dose profiles of combined radiotherapy for cervical cancer. The cervical tumor was covered with an adequately high dose, while the surrounding normal tissues including the rectum and bladder received minimum doses.

When evaluating DIR-based cumulative dose distributions and DVHs of the tumor and OARs, the accuracy of DIR is of the most importance. Although there are many uncertainties related to brachytherapy for cervical cancer [24], the accuracy of DIR can be influenced significantly by large inter-fractional variation in the organ volume and position [18,19]. In this study, therefore, pre-imaging preparations for the bladder, rectum, and uterus were performed so as to minimize such variation. In spite of preparations, in some cases, there were still variations in volume, shape and position of OARs which require some adjustment by manual procedure. This adjustment may result in variability in DIR results. With these methods, the mean DSCs for the HR-CTV, rectum, and bladder were 0.78, 0.72, and 0.81, respectively, which were significantly higher than those derived from the cumulative images by rigid fusion based on bony structure matching (Tables 1, 2 and 3). Although DSC is limited in that it does not include information of the deformation amount inside the overlapped contours, these data suggest that improved spatial overlap accuracy was obtained with the DIR method. There has been no definite consensus regarding the optimum DSC range. Kirby et al. reported that the mean DSC of the rectum was 0.85 in their study comparing 11 DIR algorithms using kilovoltage CT images of phantom [22]. Thörnqvis et al. reported that the mean DSCs of the bladder and rectum were 0.89 and 0.78, respectively, using one DIR algorithm, and 0.81 and 0.71, respectively, using another DIR algorithm [18].

Other factors which may affect the accuracy of DIR-based dose-volume evaluation are voxel size and energy conservation [25,26]. If voxel sizes of the two CT images are different, they will be normalized to the larger one during DIR process, and this process may cause some errors in cumulative dose-volume assessment. In this study, however, CT images were acquired with the same setting of field of view and the same slice thickness so as to generate same voxel size. Regarding energy conservation, the energy of radiation to one voxel may not be conserved during DIR, especially in some situation where more than one voxels which have different density are deformed to a single voxel. This may also cause some errors in DIR-based dose-volume assessment. We could not measure the energy loss with commercially available DIR software. This is another limitation of our study.

In this study, the values of the DIR-based DVH parameters were comparable to those derived from the conventional simple DVH parameter addition (Table 4). However, these results have to be carefully interpreted. According to the GEC-ESTRO recommendations, the DVH parameters for the target volumes and OARs in EBRT and each ICBT session are simply added for estimating cumulative DVH parameters. In this method, however, it has to be assumed that the location of the region of interest is identical each time [5-7]. Therefore, the cumulative DVH parameters derived from this method could be overestimated. However, there have been several reports that demonstrated positive correlation between cumulative DVH parameters derived from the simple DVH parameter addition and clinical outcomes [8-12]. It is also necessary to evaluate the correlation between DIR-based cumulative DVH parameters and clinical outcomes.

Theoretically, D2cc for OARs is an overestimation for simple DVH parameter addition, while D90 for HR-CTV is an underestimation. In our study, however, D2cc for the rectum and bladder with DIR-based DVH accumulation was higher than those with simple DVH parameter addition in one case, respectively. D90 for the HR-CTV with DIR-based DVH accumulation was lower in four cases (Table 4). Four-field box technique was used in EBRT. Inhomogeneity of EBRT doses to the uterus, rectum and bladder was within ±2% with a mean dose in a volume of interest. Therefore, inhomogeneity of dose distributions in EBRT did not greatly affect the results. One possible reason for the above results may be fusion uncertainties in DIR. In our study, we fused images and produced cumulative dose distributions for the fourth, third and second sessions of ICBT onto those of the first ICBT using DIR. During fractionated brachytherapy, most cases showed rapid diminishment of the tumors. The position and volume of the rectum and bladder also varied greatly in Case 4 despite our attempts with pre-imaging preparations. In addition, change in the volume of HRCTV cannot be reduced by pre-imaging preparation. These positional and volumetric changes may have influenced the accuracy of image fusion in DIR. Consequently, the fusion uncertainty may have resulted in the lower D90 of HR-CTV in Cases 1, 3, 4, and 5, and the higher D2cc of the rectum and bladder in Case 4. These results suggest that DIR is limited in its capacity to evaluate cumulative DVH parameters for the HR-CTV, rectum, and the bladder. However, the differences in D90 for HR-CTV and D2cc for the rectum and bladder did not differ significantly between the two methods (Table 4), and dose distributions were illustrated fairly reasonably. Therefore, we surmise that assessment of the cumulative dose-volume relationships using DIR may provide beneficial information on radiotherapy for cervical cancer, despite its limitations. For example, when a midline block is inserted into the whole pelvic EBRT, it is difficult to estimate the cumulative dose-volume relationship for the target volumes and OARs by simple addition of DVH parameters. In this case, DIR may be the only way to illustrate cumulative dose distributions and calculate cumulative DVH parameters. Furthermore, DIR may also enable to estimate the dose contributions of brachytherapy to the parametrium or pelvic lymph node. Therefore, analysis of cumulative dose-volume relationships using DIR may provide more accurate information if intensity modulated radiotherapy for cervical cancer is performed.

## Conclusions

In conclusion, though there are some limitations in accuracy of DIR, DIR-based dose accumulation may be useful method for assessing cumulative dose-volume relationship in the combined radiotherapy for cervical cancer, especially when assessing dose of the combination of midline block EBRT and brachytherapy.

## Abbreviations

EBRT: External beam radiotherapy
ICBT: Intracavitary brachytherapy
DIR: Deformable image registration
CT: Computed tomography
HR-CTV: High-risk clinical target volume
DSC: Dice similarity index
EQD2: Equivalent dose in 2 Gy per fraction
MRI: Magnetic resonance imaging
OAR: Organ at risk
DVH: Dose-volume histogram
